# Beyond the *P* Value: A Systematic Framework for Interpreting Oncology Clinical Trials

**DOI:** 10.1200/PO-26-00271

**Published:** 2026-08-13

**Authors:** Ruyue Li, Xue Dong, Xiujing Yao, Wenjie Chen, Hao An, Yintao Li

**Affiliations:** ^1^ Department of Medical Oncology, Shandong Cancer Hospital and Institute, Shandong First Medical University, and Shandong Academy of Medical Sciences, Jinan, China; ^2^ School of Clinical Medicine, Weifang Medical University, Weifang, China

## Abstract

In oncology, interpreting clinical trials solely through statistical significance (*P* < .05) often conflates true biological futility with methodological false negatives. This binary oversimplification risks prematurely abandoning active therapies while wasting resources on futile programs. We aimed to develop a structured framework to evaluate late-phase trials beyond simple *P* value assessments. Through a critical review of literature and landmark late-phase oncology trials, we analyzed common failure mechanisms and methodological pitfalls to construct a comprehensive, three-step methodology for the post hoc evaluation of negative studies. The review yielded a synthesized framework that approaches trial interpretation through three sequential steps. First, classification stratifies trials into five distinct categories: true negatives, false negatives, inconclusive trials, positive but irrelevant results, and nonsuperior but clinically valuable. Second, diagnosis uses root-cause analysis to identify underlying trial design flaws, execution biases, or statistical pitfalls. Third, recommendations outline targeted actionable strategies, including trial redesign, supplementary biomarker validation, precision medicine approaches, or scientific confirmation of therapeutic futility. This framework shifts trial interpretation from a simple win/loss assessment to a nuanced, value-based strategy. By systematically dissecting the root causes of trial failures, it empowers researchers to rescue therapies with latent clinical benefit or confidently confirm futility, thereby optimizing the evidence landscape for precision oncology.

## BACKGROUND

In oncology, well-designed clinical trials provide the high-level evidence crucial for improving patient survival and quality of life.^1^ They translate basic research into practice by evaluating novel treatments and diagnostics.^2^ Given ongoing prognostic challenges,^3^ oncology trials remain highly prevalent.

Traditionally, trial success has been defined by a *P* value <.05.^4^ Modern superiority, noninferiority, and equivalence trials no longer rely solely on *P* values; instead, they compare effect size CIs against prespecified clinical margins.^5,6^ However, the ultimate interpretation of a trial often defaults to a binary win/loss paradigm. Consequently, trials missing their primary end points are still reflexively classified as negative.^7^ However, this *P* value–driven binary evaluation is overly arbitrary.^7^ Statistical significance does not guarantee meaningful patient benefit, nor does a negative primary end point equate to biological futility. Therefore, comprehensive analyses of all end points and safety data are essential.^7^ Although investigators increasingly seek strategies to maximize the utility of complex or inconclusive trials, such efforts predominantly rely on individual empirical experience. Currently, the field lacks a standardized, structured framework to systematically manage and interpret these studies beyond their primary statistical outcomes.

**FIG 1.**
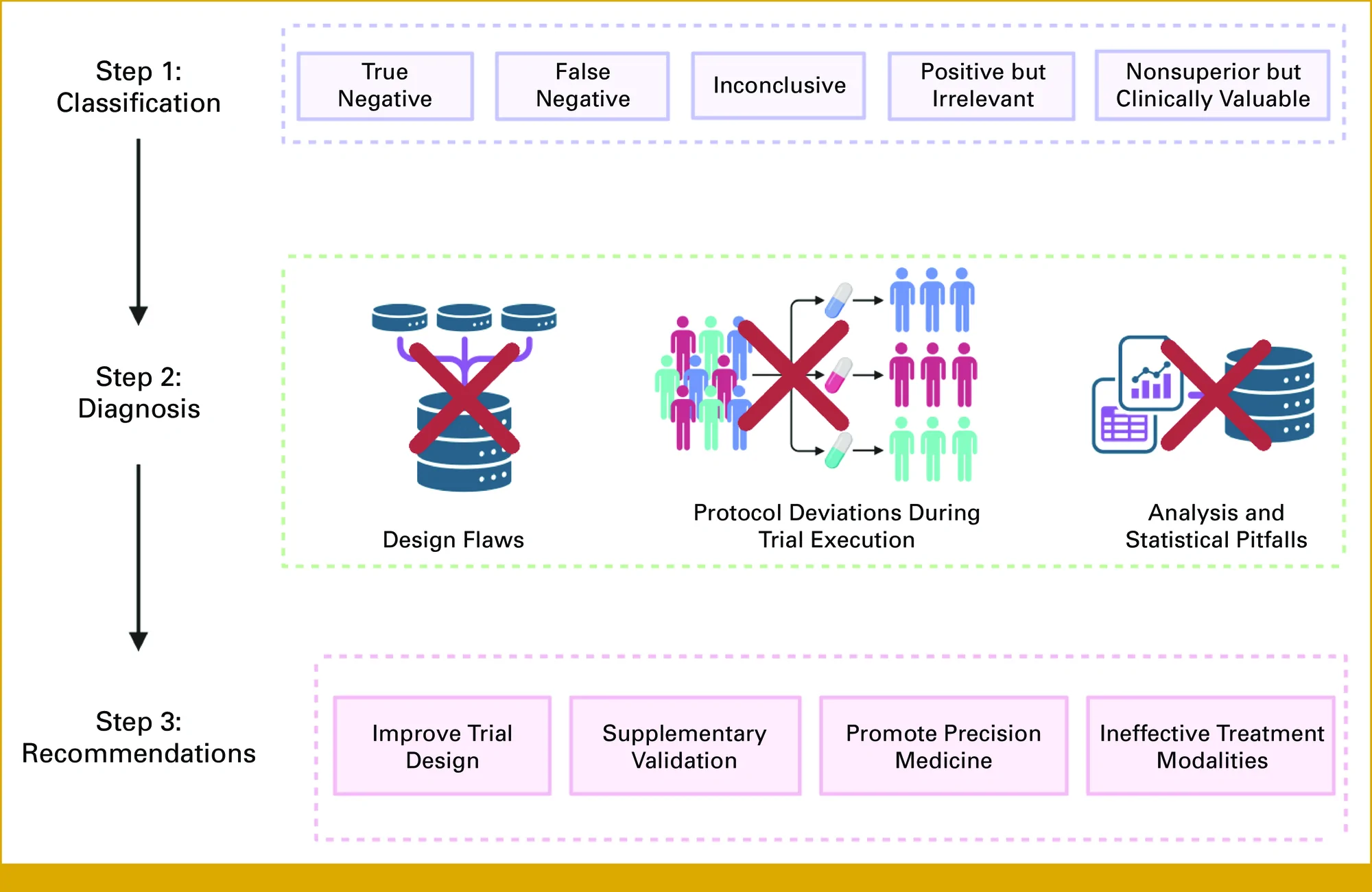
Systematic framework for interpreting negative results in clinical trials. The workflow is divided into three sequential stages. Step 1 (Classification) stratifies trial outcomes into five categories: true negative, false negative, inconclusive, positive but irrelevant, and nonsuperior but clinically valuable. Step 2 (Diagnosis) identifies the root causes of failure, such as design flaws, protocol deviations, or statistical pitfalls. Step 3 (Recommendations) outlines targeted remedial strategies, ranging from optimizing future trial designs and conducting supplementary validation to implementing precision medicine approaches or definitively ruling out ineffective modalities.

In this article, we challenge this traditional paradigm. By analyzing the causes of trial failures and ambiguous outcomes, we introduce a practical decision framework (Fig 1). This approach helps clinicians and researchers contextualize outcomes beyond the *P* value. It distinguishes true-negative failures from trials with hidden efficacy signals and evaluates the true clinical relevance of statistically positive results. This comprehensive assessment transforms statistical outcomes into valuable clinical insights, advancing precision medicine and improving patient prognosis.

## TRIAL PHASES

Translating basic research into clinical oncology requires rigorous trial designs that sequentially validate safety, dosing, and efficacy. Understanding the distinct objectives and failure mechanisms of each trial phase is essential for establishing an evaluation framework that moves beyond simple *P* value interpretations.

Phase I trials primarily establish safety and dosing. Negative outcomes here typically stem from intrinsic pharmacological flaws, such as unacceptable toxicity or insufficient target engagement.^8,9^ For instance, a phase Ib trial of the bromodomain and extra-terminal (BET) inhibitor RO6870810 plus atezolizumab was terminated due to severe immune-related toxicity and minimal efficacy (5.6% objective response rate [ORR]) despite target engagement.^8^ Addressing such early failures requires biological adjustments, as post hoc statistical manipulations are generally uninformative.^10^

Building on these safety data, phase II trials preliminarily validate efficacy using surrogate end points like ORR and progression-free survival (PFS) to determine the feasibility of phase III studies. Although failing to meet the primary end point defines a negative phase II trial, latent clinical benefit may still exist. For example, a phase II trial evaluating nivolumab plus ipilimumab in cancer of unknown primary missed its overall ORR end point (16%). However, subsequent analysis revealed a robust 60% ORR in the tumor mutational burden (TMB)-high cohort versus 7.7% in the TMB-low cohort.^11^ This underscores the need for a framework to extract valid biological signals beyond superficial statistical failures.

Confirmatory phase III trials evaluate therapies against the standard of care using robust end points like overall survival (OS) and PFS. Statistically negative results here may reflect statistical confounding rather than biological futility. For instance, in the phase III CheckMate-026 trial of first-line nivolumab for advanced non–small cell lung cancer (NSCLC), the failure to improve PFS in the PD-L1 ≥ 5% cohort (4.2 *v* 5.9 months; hazard ratio [HR], 1.15 [95% CI, 0.91 to 1.45]; *P* = .25), combined with the confounding effect of a 60% crossover rate on OS (14.4 *v* 13.2 months; HR, 1.02 [95% CI, 0.80 to 1.30]), suggests that patient selection and trial design drove the negative outcome.^12^ Therefore, a structured evaluation framework is imperative to retrospectively disentangle these methodological pitfalls from true biological futility.

Although early-phase failures are predominantly pharmacological, randomized late-phase trials are frequently misinterpreted due to patient heterogeneity, rigid statistical thresholds, and confounding factors. Given their profound implications for clinical practice, this article focuses on applying a structured framework to late-phase trials that missed their primary end points, with the aim of systematically identifying the underlying causes and uncovering their latent therapeutic value.

## STEP 1: CLASSIFICATION

**FIG 2.**
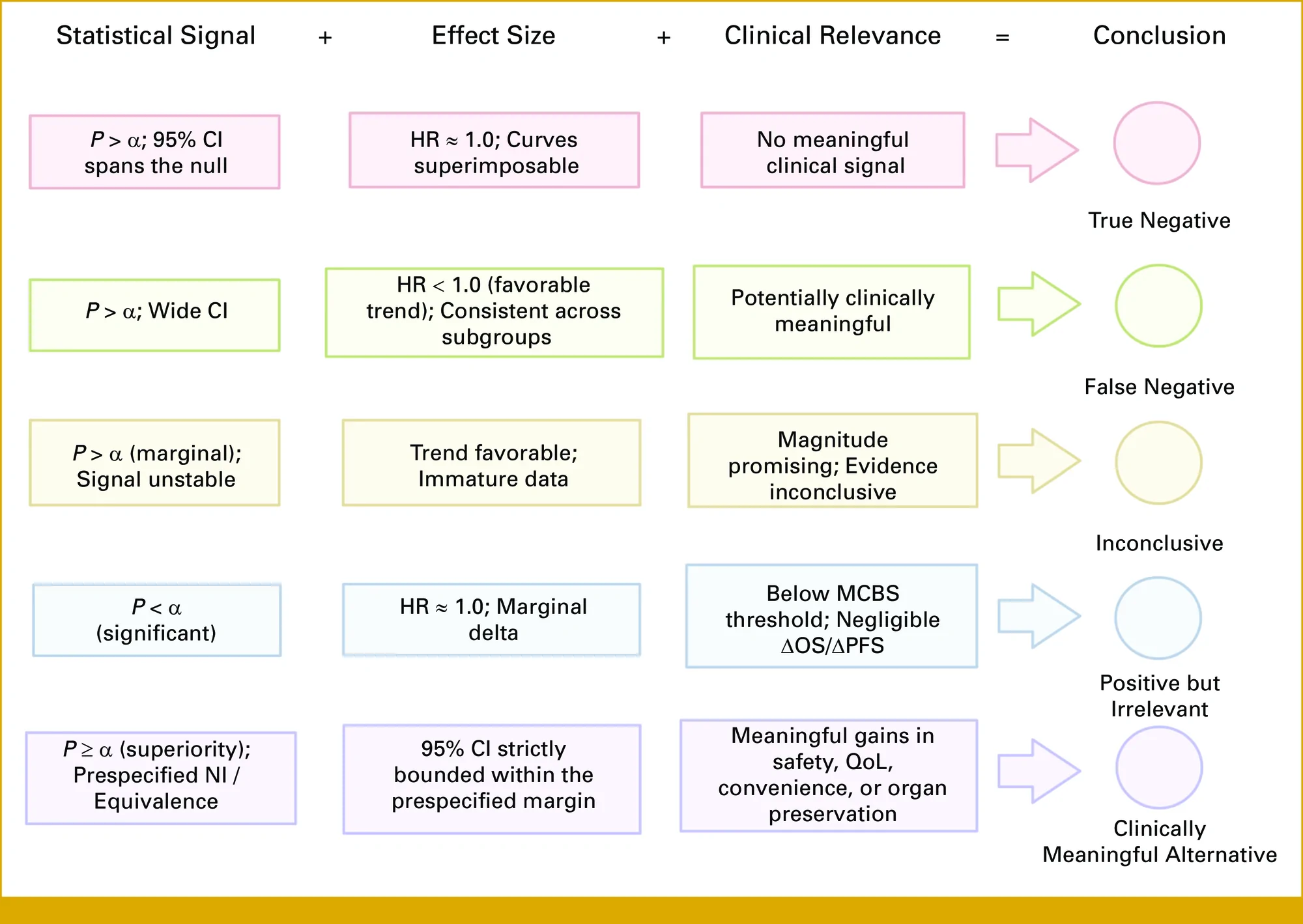
Multidimensional framework for interpreting trial outcomes. This framework illustrates how integrating statistical signals, effect size, and clinical relevance determines the true nature of a trial result. Although True Negative represents a clear lack of efficacy, False Negative identifies scenarios where a favorable effect size is masked by wide CIs. Inconclusive results reflect immature data, whereas Positive but Irrelevant highlights the critical distinction between statistical significance and a lack of meaningful clinical benefit. Importantly, nonsuperior but clinically valuable outcomes demonstrate that in prespecified noninferiority or equivalence trials, treatments can yield a positive result if the CI falls strictly within the predefined margin and provides patient-centered advantages. HR, hazard ratio; MCBS, magnitude of clinical benefit scale; NI, noninferiority; OS, overall survival; PFS, progression-free survival; QoL, quality of life.

Rigorous interpretation of negative trials is pivotal for evidence-based medicine. We establish a framework to classify these trials, facilitating the rapid assessment of their intrinsic value across five primary dimensions (Fig 2).

### True Negative

Well-designed, rigorous, and adequately powered trials that fail to achieve statistical significance on primary end points are classified as true-negative trials, validating the null hypothesis. In the NSCLC landscape, several robust studies have returned true-negative findings. For example, the phase III KEYNOTE-598 trial^13^ evaluating the addition of ipilimumab to pembrolizumab in treatment-naïve metastatic NSCLC (PD-L1 tumor proportion score ≥50%) showed no improvement in OS (21.4 *v* 21.9 months; HR, 1.08 [95% CI, 0.85 to 1.37]; *P* = .74) or PFS (8.2 *v* 8.4 months; HR, 1.06 [95% CI, 0.86 to 1.30]; *P* = .72), alongside increased toxicity. Similarly, the phase III PACIFIC-2 trial^14^ evaluating durvalumab added to concurrent chemoradiotherapy in unresectable stage III NSCLC demonstrated no benefit in PFS (HR, 0.85; *P* = .247) or OS (HR, 1.03; *P* = .823). Additionally, trials across renal cell carcinoma (RCC), melanoma, and head and neck squamous cell carcinoma (HNSCC), including IMmotion010,^15^ CheckMate 914,^16^ CheckMate 915,^17^ and IMvoke010,^18^ reported no significant clinical benefit. These true-negative results are not failures. Instead, they characterize therapeutic limitations within specific biological contexts, identify ineffective strategies, and ensure the redirection of resources and research focus toward scientifically rational avenues.

### False Negative

Trials in which an objectively effective therapy fails to achieve statistical significance on the primary end point due to flaws in design, execution, or analysis are defined as false-negative trials. Notably, many of those trials still possess clinical utility. For instance, the LEAP-002 study^19^ in first-line hepatocellular carcinoma (HCC) reported a median OS of 21.2 months for lenvatinib plus pembrolizumab versus 19 months for lenvatinib alone (HR, 0.84; *P* = .023), missing the prespecified significance threshold of *P* = .019. Similarly, the KEYNOTE-789 trial^20^ in previously treated epidermal growth factor receptor (*EGFR*)–mutant NSCLC demonstrated a trend toward OS benefit (HR, 0.84 [95% CI, 0.69 to 1.02]; *P* = .0362) but did not meet the statistical threshold (*P* = .0117). As similar false-negative outcomes driven by heterogeneous factors occur across tumor types, comprehensive analyses are essential to uncover underlying causes. Converting these insights into actionable improvements will optimize future patient selection and trial design, accelerating the generation of translatable evidence.

### Inconclusive

Not all trials failing to meet primary end points are futile. Inconclusive trials typically demonstrate a trend toward benefit but lack statistical significance. The long-tail effect of immunotherapy is a classic example.^21^ For instance, in the CheckMate 816 study^22^ of neoadjuvant nivolumab plus chemotherapy for resectable NSCLC, the first interim OS analysis missed the significance threshold (HR, 0.57 [99.67% CI, 0.30 to 1.07]); however, the final analysis confirmed significant OS improvement (HR, 0.72 [95% CI, 0.523 to 0.998]; *P* = .048; 5-year OS, 65.4% *v* 55%).^23^ Similarly, the phase III CheckMate 026 trial^12^ showed that first-line nivolumab was not superior to standard chemotherapy in advanced or recurrent NSCLC with PD-L1 ≥ 5% (*P* = .25), yet Kaplan-Meier curves revealed a delayed treatment effect. Additionally, some trials miss primary statistical end points but demonstrate clear clinical benefit in specific biomarker-defined subgroups. In the phase III IMvigor010 study,^24^ adjuvant atezolizumab missed overall disease-free survival (DFS) in muscle-invasive urothelial carcinoma (19.4 *v* 16.6 months; HR, 0.89 [95% CI, 0.74 to 1.08]; *P* = .24). Yet, exploratory analysis revealed significant DFS (HR, 0.58 [95% CI, 0.43 to 0.79]; *P* = .0024) and OS (HR, 0.59) benefits solely in circulating tumor DNA (ctDNA)–positive patients.^25^ This confirms ctDNA as a predictive marker for adjuvant immunotherapy to prevent overtreatment. Accurately identifying such causes and refining trial designs holds promise for reversing apparent clinical trial failures.

### Positive but Irrelevant

Clinical trials that meet primary statistical end points but fail to demonstrate real-world benefit can be considered clinically futile despite statistical success. One primary cause is the failure of surrogate end points, such as PFS, to translate into long-term survival. In the phase III COSMIC-312 trial^26^ for advanced HCC, cabozantinib plus atezolizumab significantly improved PFS versus sorafenib (median, 6.8 *v* 4.2 months; HR, 0.63 [99% CI, 0.44 to 0.91]; *P* = .0012). However, this combination provided no OS advantage (median, 15.4 *v* 15.5 months; HR, 0.90 [96% CI, 0.69 to 1.18]; *P* = .44). Phase III trials ATTRACTION-4^27^ and PROpel^28^ demonstrated similar discordance. Furthermore, trials may achieve statistical significance while demonstrating negligible absolute benefit that fails to meet the minimal clinically important difference. In the phase III IPSOS trial^29^ for advanced NSCLC, first-line atezolizumab significantly improved OS versus chemotherapy (HR, 0.78 [95% CI, 0.63 to 0.97]; *P* = .028); however, the median OS extension was merely 1.1 months, reflecting limited clinical utility. NAPOLI-3^30^ and KEYNOTE-859^31^ exhibited similar marginal gains. Consequently, a positive *P* value alone does not confirm clinical efficacy. Tools like the European Society for Medical Oncology Magnitude of Clinical Benefit Scale and the ASCO Value Framework are crucial for properly contextualizing these trials.

### Nonsuperior but Clinically Valuable

Noninferiority and equivalence trials do not require superiority. Rather, they rely on CIs and prespecified margins to confirm comparable efficacy, often highlighting secondary advantages like safety, convenience, or quality of life. For example, a phase III trial in locally advanced nasopharyngeal carcinoma^32^ compared radiotherapy volumes before and after induction chemotherapy. The postchemotherapy volume plan maintained a comparable 3-year survival rate but significantly reduced severe toxicities via a smaller irradiation field, establishing a safer option for frail patients. Similarly, the SANO trial^33^ demonstrated that active surveillance following neoadjuvant chemoradiotherapy for esophageal cancer was noninferior to standard esophagectomy regarding 2-year survival (75% [95% CI, 68 to 80] *v* 70% [95% CI, 63 to 77]), sparing selected patients from high-risk surgery. Furthermore, equivalence trials confirm comparable efficacy, such as the LILAC trial demonstrating equivalent pathological complete response rates between the biosimilar ABP 980 and originator trastuzumab in human epidermal growth factor receptor 2–positive breast cancer,^34^ and the SABRINA trial establishing comparable efficacy and safety for subcutaneous versus intravenous rituximab.^35^

## STEP 2: DIAGNOSIS

**FIG 3.**
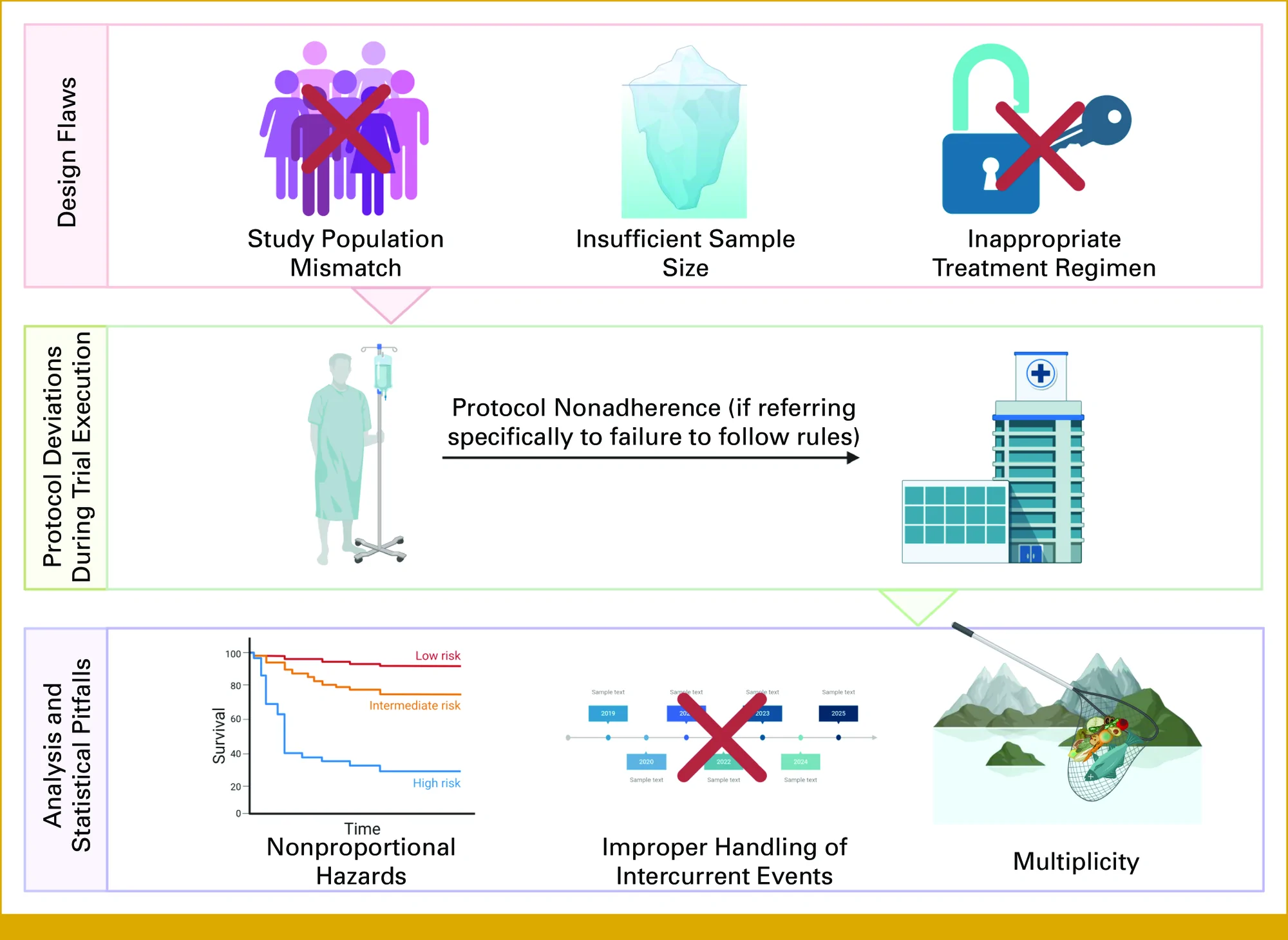
Specific causes of negative clinical trial results. The figure delineates three major categories of errors contributing to negative trial outcomes: (1) Design Flaws (eg, population mismatch, insufficient sample size, or inappropriate treatment regimen); (2) Execution Deviations (eg, protocol nonadherence); and (3) Statistical Pitfalls (eg, violation of proportional hazards assumption or multiplicity issues).

Following initial classification, we identify the root causes of negative or inconclusive trials, which may stem from any phase of the trial lifecycle (Fig 3).

### Design Flaws

#### Study Population Mismatch

Patient heterogeneity can easily mask significant treatment effects present only in specific subgroups. For instance, after gefitinib initially failed in unselected patients with lung cancer, a subgroup analysis of the IPASS study revealed a significant benefit in patients with *EGFR* mutations.^36^ Similarly, identifying precise predictive biomarkers is critical for immunotherapy. The JAVELIN Lung 200 trial^37^ initially failed to show a survival benefit for avelumab over docetaxel in patients with advanced NSCLC and broad PD-L1 positivity (≥1%) (median OS, 11.4 *v* 10.3 months). However, subsequent analysis demonstrated that in subgroups with high PD-L1 expression (≥50% and ≥80%), the 2-year survival rate with avelumab nearly doubled that of chemotherapy.^38^ Furthermore, ethnic and biological disparities affect outcomes; the IMpower151 trial^39^ in Chinese patients with NSCLC failed to replicate the positive efficacy of its immune combination therapy observed in global populations.

#### Insufficient Sample Size

Inadequate sample sizes often precipitate type II errors (false negatives). The ATALANTE-1 trial, evaluating OSE2101 in immunotherapy-resistant advanced NSCLC, planned for 363 patients but terminated prematurely due to the COVID-19 pandemic with only 219 randomly assigned. Consequently underpowered, the trial failed to demonstrate a significant survival benefit for OSE2101 over the standard of care (HR, 0.86; *P* = .36).^40^ This highlights how external disruptions leading to underenrollment directly compromise trial validity.

#### Inappropriate Treatment Regimen

Flawed drug combinations, dosing, or sequencing can derail trials. Combining PD-1/PD-L1 inhibitors with cytotoxic T lymphocyte–associated antigen-4 inhibitors typically yields higher toxicity than monotherapy.^41^ In the CheckMate-714 (HNSCC) and CheckMate-915 (melanoma) trials, adding ipilimumab to nivolumab failed to improve ORRs but significantly increased treatment-related adverse events.^17,42^ Conversely, rational combinations yield synergy, as demonstrated by KEYNOTE-189, where first-line pembrolizumab plus pemetrexed-platinum chemotherapy significantly improved both OS and PFS in metastatic nonsquamous NSCLC.^43^

### Protocol Deviations During Trial Execution

A benchmark study (2013-2019) revealed that oncology trials experienced nearly 20% more protocol deviations than nononcology trials, affecting roughly one third of enrolled patients.^44^ Trial execution quality is a critical factor in interpreting negative clinical trial results. According to Food and Drug Administration guidance, major protocol deviations include poor patient compliance, treatment discontinuation, and missing data. These can dilute the actual treatment effect, leading a potentially effective therapy to be incorrectly deemed ineffective—a false negative. For instance, in the PORT-C trial, the evaluation of postoperative radiotherapy (PORT) in stage IIIA-N2 NSCLC was negative (median DFS, *P* = .20). However, 23.9% of the patients refused to undergo PORT. A subsequent per-protocol analysis showed that adjuvant PORT significantly improved the 3-year DFS rate (42.8% *v* 30.6%; *P* = .05).^45^ Similarly, the Radiation Therapy Oncology Group 9704 (pancreatic cancer) and CAO/ARO/AIO-04 (rectal cancer) trials demonstrated compromised outcomes in nonadherent patients, underscoring the necessity of evaluating execution quality when interpreting negative trials.^46,47^

### Analysis and Statistical Pitfalls

#### Nonproportional Hazards

Standard survival analyses, including the log-rank test and Cox model, rely on the proportional hazards (PH) assumption of a constant HR over time. However, immunotherapies frequently exhibit delayed clinical benefits. In the phase III CheckMate 026 trial, first-line nivolumab failed to show superiority over chemotherapy in advanced NSCLC (PD-L1 ≥ 1%), yet Kaplan-Meier curves indicated a delayed treatment effect, rendering a single HR inadequate.^12^ To address non-PH scenarios, alternative tools like restricted mean survival time and weighted log-rank tests are essential.

#### Improper Handling of Intercurrent Events

Failure to appropriately adjust for intercurrent events, notably treatment crossover, can severely confound survival analyses. In the VEG105192 study evaluating pazopanib for advanced RCC, the intention-to-treat (ITT) analysis showed no OS benefit (22.9 *v* 20.5 months; HR, 0.91 [95% CI, 0.71 to 1.16]; *P* = .224) due to a 51% placebo-to-pazopanib crossover rate after progression. After adjusting for crossover using the Inverse Probability of Censoring Weighting (IPCW) method, pazopanib demonstrated a significant reduction in mortality risk (HR, 0.504 [95% CI, 0.315 to 0.762]; *P* = .002), confirming its true efficacy.^48^

#### Multiplicity Testing

Multiplicity testing increases the probability of a false-positive finding (a type I error). For example, in the IMpassion130 trial of atezolizumab for first-line metastatic triple-negative breast cancer (TNBC), the alpha level for OS was prespecified at 0.04 to control for this error. Accordingly, the OS in the ITT population did not achieve statistical significance (*P* = .08).^49^

## STEP 3: RECOMMENDATIONS

**FIG 4.**
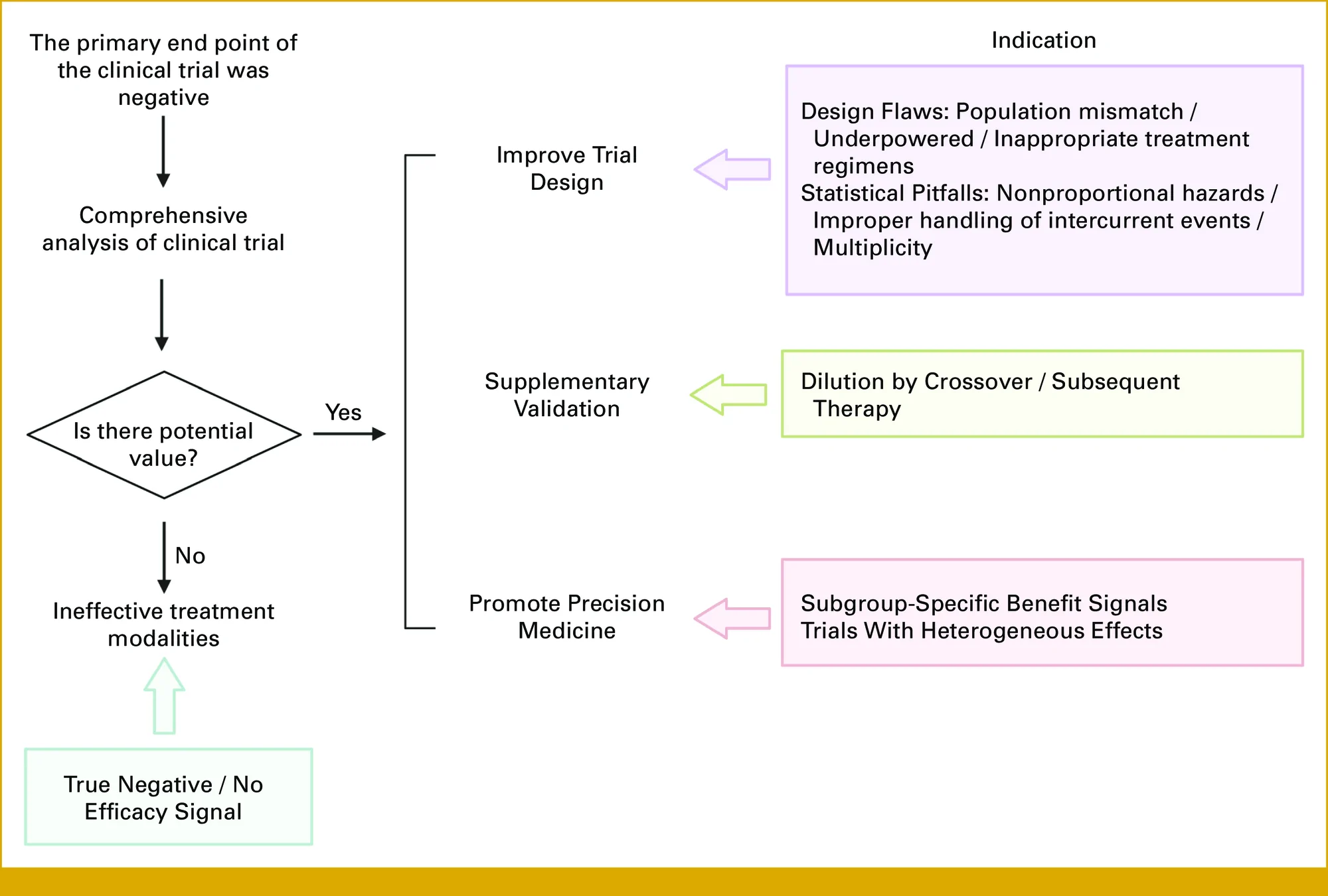
Decision framework for the post hoc management of negative clinical trials. The flow chart outlines a structured approach to evaluating trials that fail to meet their primary end points. Following a comprehensive analysis, outcomes are triaged based on their potential value. Improve Trial Design is recommended when failures stem from methodological flaws or statistical artifacts. Supplementary Validation is advised when efficacy signals are diluted by confounding factors, such as crossover or subsequent therapies. Promote Precision Medicine is indicated when heterogeneous treatment effects suggest subgroup-specific benefits. Ineffective Treatment Modalities is reserved for trials with clear true-negative signals.

By diagnosing the specific underlying causes contributing to unmet primary end points in oncology trials, we propose the following actionable pathways (Fig 4).

### Improve Trial Design

Successful trial design iteratively builds on foundational research. However, translating preclinical rationale into late-stage success is challenged by patient heterogeneity and methodological flaws. Consequently, we established an optimization pathway for trials with design flaws or statistical limitations. When pivotal late-stage trials fail, investigators should comprehensively evaluate the underlying causes via post hoc analyses rather than repeating the design in unselected populations. These analyses aim to identify predictive biomarkers or justify exploratory combination therapies, guiding future protocols using biomarker-driven enrichment or adaptive statistical methods. For clinicians, major guidelines mandate restricting off-label use to settings lacking standard-of-care options. Evidence derived solely from subgroup analysis requires extreme caution; routine recommendation is discouraged unless the subgroup effect is highly credible (eg, prespecified, biologically plausible), clinically meaningful (eg, HR < 0.6-0.7), and offers a favorable risk-benefit ratio.^50^

The KEYNOTE-119 study exemplifies this iterative improvement. The phase III KEYNOTE-119 trial^51^ evaluating pembrolizumab monotherapy versus chemotherapy in pretreated metastatic TNBC missed its primary OS end point (median OS, 9.9 *v* 10.8 months). Subsequently, the KEYNOTE-355 design was optimized: in patients with a PD-L1 combined positive score ≥10, the median PFS for pembrolizumab plus chemotherapy versus placebo plus chemotherapy significantly improved (9.7 *v* 5.6 months; one-sided *P* = .0012).^52^ Building on this, KEYNOTE-522 demonstrated significantly higher pathological complete response rates for neoadjuvant pembrolizumab plus chemotherapy in early TNBC versus placebo (64.8% *v* 51.2%; *P* < .001).^53^ Additionally, Cortes et al^54^ predicted the response of metastatic TNBC to pembrolizumab monotherapy by quantifying PD-L1 expression across various cell types.

### Supplementary Validation

Ethical considerations often mandate allowing control arm patients to cross over to experimental therapies upon progression. Although this may prolong OS, the resulting dilution effect can yield false-negative ITT analyses. Elucidating true therapeutic efficacy requires proper upfront trial design rather than subsequent statistical salvage.

Post hoc statistical adjustments cannot compensate for fundamentally flawed designs. Rather than inefficiently inflating sample sizes, investigators must anticipate crossover dynamics during design. Per the ICH E9(R1) framework,^55^ strategies for handling intercurrent events such as treatment crossover must be prespecified in the statistical analysis plan. Post hoc crossover-adjusted analyses are inherently exploratory, as the prevalence of treatment crossover in oncology trials is high, and the timing and implementation of crossover further influence the assignment of evidence levels by regulatory and Health Technology Assessment agencies.^56,57^ Such analyses should generate hypotheses for future prospectively designed studies rather than serve as the primary basis for approval. When supporting regulatory decisions, counterfactual adjustment methods (eg, IPCW, Rank-Preserving Structural Failure Time Model, two-stage estimation) must be prespecified in the statistical analysis plan^58^; otherwise, they fail evidentiary standards.^59^ Clinicians and regulators do not reject a treatment solely based on nonsignificant ITT *P* values if the analysis incorporates biological plausibility and prespecified crossover adjustments.^60^ The phase III PROfound trial^61^ illustrates this. Evaluating olaparib versus control in metastatic castration-resistant prostate cancer, the trial ethically permitted a 67% crossover rate. Although the primary OS benefit was significant (HR, 0.69), its magnitude was dampened. Applying the prespecified IPCW method to adjust for crossover, olaparib reduced mortality risk by 58% (HR, 0.42 [95% CI, 0.19 to 0.91]) versus the adjusted control. Conversely, the phase III CodeBreaK 200 study,^62^ comparing sotorasib with docetaxel in *KRAS* G12C-mutated NSCLC, met its primary PFS end point (5.6 [95% CI, 4.3 to 7.8] *v* 4.5 months [95% CI, 3 to 5.7]; HR, 0.66 [95% CI, 0.51 to 0.86]; *P* = .0017) but showed no significant OS difference (HR, 1.01 [95% CI, 0.77 to 1.33]). This was likely driven by reduced sample size and permitted crossover.^63^ Both studies underscore that prespecifying adjustment strategies is essential for unmasking true clinical benefit.

### Promote Precision Medicine

Subgroup analysis of negative trials can uncover hidden efficacy and advance precision medicine by exploring tumor heterogeneity. Prospective subgroup analysis identifies beneficiaries, whereas exploratory post hoc analysis generates new hypotheses. We established an optimization pathway for trials where heterogeneity masks efficacy.

The interpretability of subgroup analyses depends on prespecification and statistical rigor. Prespecified subgroups based on established mechanisms carry the highest evidence level. With appropriate multiple testing correction, significant interaction tests and consistent secondary end points provide robust evidence. Conversely, post hoc analyses only generate hypotheses requiring independent validation.

Clinicians must interpret subgroup data from underpowered negative trials cautiously due to inflated false-positive and false-negative risks. Rather than relying solely on conservative Bonferroni corrections, incorporating Bayesian models or the false discovery rate scientifically balances these risks. Subgroup results from a negative trial hold clinical value only when meeting three criteria: (1) strong biological plausibility; (2) consistent interaction trends (even if nominal *P* values fail, clear benefit in the target subgroup versus neutral effect in controls, with meaningful CIs, remains highly suggestive); and (3) clinically meaningful effect sizes. Routine treatment decisions must never be altered based solely on unvalidated post hoc analyses.

The ICON7 trial exemplifies prespecified subgroup utility. Despite no general OS benefit for bevacizumab in ovarian cancer (44.6 *v* 45.5 months; *P* = .85), prespecified analysis identified a significant OS advantage in the high-risk subgroup (*P* = .03).^64^ Additionally, several studies have shown that exploratory analyses can reveal latent efficacy signals and pinpoint the specific target population. For example, in the IMpassion130 trial, no significant difference in median OS was observed between patients with unresectable TNBC treated with atezolizumab versus placebo (21 *v* 18.7 months; HR, 0.86; *P* = .078). However, a post hoc analysis revealed a clinically meaningful OS benefit in the PD-L1–positive subgroup (HR, 0.71 [95% CI, 0.54 to 0.94]; *P* value not formally tested).^65^ Similarly, the JAVELIN Lung 200 trial of avelumab versus docetaxel in pretreated NSCLC showed no overall OS difference (11.4 *v* 10.3 months; HR, 0.90; one-sided *P* = .16).^37^ Yet, exploratory analysis uncovered a significant survival benefit in high PD-L1 expressors. Therefore, rather than relying on strict statistical significance, hypothesis-generating exploratory analyses can advance oncology research by identifying meaningful clinical signals, such as favorable effect sizes. Because the *P* values in these analyses are not adjusted for multiple comparisons, they are nominal and serve only as a reference. However, multiple testing correction is typically not recommended in this context to avoid increasing the type II error rate, which could mask efficacious subgroups. Consequently, these findings must be interpreted with caution and should be used solely as a rationale for conducting subsequent large-scale, prospective confirmatory trials. Furthermore, exploratory analyses of the negative AVAglio and PRINCE trials identified the *IDH1* wild-type proneural subtype and a unique immune signature as predictive biomarkers, respectively.^66-68^ These frontier discoveries provide pivotal research directions for future clinical trials.

### Ineffective Treatment Modalities

True-negative trials are rigorously designed studies that fail to meet primary end points with negligible effect sizes (eg, HR ≈ 1) and no reliable subgroup benefit. Rather than being failures, these trials represent crucial scientific progress.^69^ The most scientific approach for such trials is to promptly conclude the study and transparently share the findings to overcome publication bias. This practice prevents duplicate futile trials, helps clinicians avoid ineffective off-label combinations, and protects patients from unnecessary toxicity.

The phase III ECHO-301/KEYNOTE-252 trial^70^ demonstrated this. Despite promising early-phase data, the confirmatory trial showed absolutely no improvement in PFS or OS compared with pembrolizumab alone (HR, 1). Similarly, the EVOLVE-1 trial^71^ evaluated everolimus in HCC after sorafenib failure. Everolimus did not improve OS, yielding mortality comparable with placebo (83.7% *v* 82.1%), definitively proving that singular mammalian target of rapamycin inhibition is ineffective here. Additionally, a phase Ib study evaluating the BET inhibitor RO6870810 combined with atezolizumab was terminated early due to severe safety concerns and a very low response rate of only 5.6%.^8^

## CLINICAL IMPLICATIONS AND FUTURE DIRECTIONS

Interpreting trials through our structured framework positively affects oncology research by providing a holistic evidence assessment. It enables nuanced recommendations: formally rejecting futile interventions, suggesting specific subgroup usage pending prospective validation, and positioning treatments with benefit trends as alternatives.

As illustrated in Table 1, applying this framework yields distinct, actionable pathways. For true-negative trials,^72-74^ it provides the confidence to de-implement futile interventions, sparing patients from overtreatment. Conversely, for inconclusive or suspected false-negative results,^75,76^ it prevents the premature abandonment of potentially active therapies, generating targeted hypotheses for subsequent prospective validation.

**TABLE 1.** Translating the Framework Into Practice: Deconstructing Trials Beyond the *P* Value

Case A: Accurately Identifying True-Negative Results to Avoid Overtreatment	
Background	The STORM trial^72^ investigated adjuvant sorafenib versus placebo following curative resection or ablation for hepatocellular carcinoma. The primary end point, recurrence-free survival, was not improved (33.3 *v* 33.7 months; HR, 0.94 [95% CI, 0.78 to 1.13]; one-sided *P* = .26), and there was no OS benefit (*P* = .48)
Step 1 (Classification)	True negative
Step 2 (Diagnosis)	Multiple studies have demonstrated that multikinase and antiangiogenic therapies offer limited clinical benefit in the adjuvant setting.^73,74^ Therefore, this true-negative result is not attributable to trial design flaws
Step 3 (Recommendations)	De-implement and redirect. Confirm futility and avoid off-label use of adjuvant sorafenib in routine practice. Clinical and translational resources should be redirected toward alternative adjuvant strategies supported by stronger mechanistic rationales and robust early-phase efficacy signals

Case B: Selection Bias Leading to False Negative or Inconclusive Results	
Background	In the GOG-0213 trial,^75^ secondary cytoreductive surgery did not improve OS compared with chemotherapy alone in patients with platinum-sensitive recurrent ovarian cancer (50.6 *v* 64.7 months; HR, 1.29 [95% CI, 0.97 to 1.72]; *P* = .08). Notably, complete gross resection was achieved in 67% of patients
Step 1 (Classification)	False negative/inconclusive
Step 2 (Diagnosis)	The overall negative outcome is highly likely attributable to unstandardized surgical indications, which introduced significant patient heterogeneity
Step 3 (Recommendations)	Refine and validate. Rather than abandoning the surgical strategy in light of this apparent failure, investigators should focus on predictive enrichment. This approach was successfully validated in the subsequent DESKTOP III trial, which restricted enrollment using standardized criteria (the AGO score) and confirmed a significant OS benefit (53.7 *v* 46 months; HR, 0.75 [95% CI, 0.59 to 0.96]; *P* = .02)^76^

NOTE. To bridge the gap between theoretical frameworks and real-world clinical practice, we applied our three-step approach to two challenging trials. Notably, although both trials failed to meet their primary end points, applying this framework led to starkly different, yet highly actionable, clinical conclusions.

Abbreviations: AGO, Arbeitsgemeinschaft Gynäkologische Onkologie; HR, hazard ratio; OS, overall survival.

## IMPROVING STATISTICAL METHODS AND REPORTING TRANSPARENCY

To accelerate progress, we advocate for a new research ecosystem for trial methodology and reporting. Key elements include: (1) clear objectives and prespecified handling of intercurrent events; (2) reporting effect sizes and CIs for all end points; (3) complete reporting of subgroup analyses; (4) transparent multiplicity control strategies; and (5) full data sharing. Standardization is essential to maximize research iteration and drive rapid advances in cancer care.

## DISCUSSION

Oncology trials failing to meet statistical significance are not failures but indispensable components of the research process. To address current interpretative gaps, we propose a structured framework comprising Classification, Diagnosis, and Recommendations. This framework translates fragmented experiences into actionable guidelines, standardizes post hoc workflows to minimize analytical bias, and democratizes expert insights for all oncology professionals. By standardizing the post-trial diagnostic trajectory, it optimizes the translational value of negative trials in precision oncology.

To achieve this, we urge researchers and sponsors to transparently report all outcomes, evaluating evidence holistically. Furthermore, high-impact journals must champion the publication of well-conducted negative trials. Embracing these complex outcomes is essential to advance precision oncology and ultimately improve patient survival.

## Data Availability

This manuscript is a literature review and did not generate any new primary data. All data analyzed and discussed in this study are sourced from previously published research. These original sources are fully cited in the text and listed in the reference section. Interested readers may refer to the original publications to access the data, subject to the policies of the respective journals and authors.
